# Identification of Mycoviruses in the Pathogens of Fragrant Pear Valsa Canker from Xinjiang in China

**DOI:** 10.3390/v16030355

**Published:** 2024-02-25

**Authors:** Chenguang Zhang, Xiaoya Zheng, Zhanjiang Tie, Hui Xi, Mai Shi, Yanjun Ma, Wenbin Chen, Yingjie Mi, Rui Yang, Sifeng Zhao, Xuekun Zhang

**Affiliations:** 1 Key Laboratory of Oasis Agricultural Pest Management and Plant Protection Resources Utilization, College of Agriculture, Xinjiang Uygur Autonomous Region, Shihezi University, Shihezi 832003, China; zcg8888666@163.com (C.Z.); 18083946009@163.com (X.Z.); t2424395514@163.com (Z.T.); xihui@shzu.edu.cn (H.X.); 18875761735@163.com (M.S.); 13899014867@163.com (Y.M.); 15955651230@163.com (W.C.); 15630077211@163.com (Y.M.); 2 Agricultural Technology Extension Station of the First Division, Alaer 843300, China; 13779808226@163.com

**Keywords:** mycovirus, metatranscriptomics, fragrant pear canker disease, biocontrol, *Valsa* spp.

## Abstract

As a common disease, canker seriously affects the yield and quality of fragrant pear due to the lack of effective control measures. Some fungi have been reported to harbor rich reservoirs of viral resources, and some mycoviruses can be used as biocontrol agents against plant diseases. In this study, 199 isolates were obtained from diseased branches of fragrant pear in the main production areas of Xinjiang. Among them, 134 belonged to *Valsa* spp., identified using morphological and molecular biological techniques, in which *V. mali* was the dominant species. The mycoviruses in *Valsa* spp. were further identified using metatranscriptomic sequencing and RT-PCR. The results revealed that a total of seven mycoviruses were identified, belonging to *Botourmiaviridae*, *Endornaviridae*, *Fusariviridae*, *Hypoviridae*, *Mitoviridae*, and *Narnaviridae*, among which Phomopsis longicolla hypovirus (PlHV) was dominant in all the sample collection regions. The Cryphonectria hypovirus 3-XJ1 (CHV3-XJ1), Botourmiaviridae sp.-XJ1 (BVsp-XJ1), and Fusariviridae sp.-XJ1 (Fvsp-XJ1) were new mycoviruses discovered within the *Valsa* spp. More importantly, compared with those in the virus-free *Valsa* spp. strain, the growth rate and virulence of the VN-5 strain co-infected with PlHV and CHV3-XJ1 were reduced by 59% and 75%, respectively, and the growth rate and virulence of the VN-34 strain infected with PlHV were reduced by 42% and 55%, respectively. On the other hand, the horizontal transmission efficiency of PlHV decreased when PlHV was co-infected with CHV3-XJ1, indicating that PlHV and CHV3-XJ1 were antagonistic. In summary, the mycoviruses in *Valsa* spp. were identified in Xinjiang for the first time, and three of them were newly discovered mycoviruses, with two strains yielding good results. These results will offer potential biocontrol resources for managing pear canker disease and provide a theoretical basis for the control of fruit tree Valsa canker disease.

## 1. Introduction

‘Korla’ fragrant pear (*Pyrus sinkiangensis* Yü), known as “China’s honey pears” and the “prince of fruits”, is a traditional cultivar that is mainly grown around the Korla region of Xinjiang Province in Western China [1]. Fragrant pear has a long history of cultivation and good fruit quality, and it occupies an important position in Xinjiang and even worldwide. The rapid development of the fragrant pear industry has made it a crucial pillar of the regional fruit industry, with important economic and strategic value. However, Valsa canker causes significant yield losses globally, particularly in regions such as East Asia, China, Italy, and other regions, where it poses a major threat to pear fruit production, often resulting in reduced yields or tree mortality in entire orchards [2,3]. At present, diseased spots are extensively scraped away, and large amounts of pesticides are applied to diseased spots to control this disease [4]. Currently, the recurrence rate of Valsa canker lesions remains high, and there is a lack of dedicated pesticides for effective control in fragrant pear trees. *Bacillus atrophaeus* and *B. vallismortis* T27 should be considered for use as biocontrol agents for controlling pear Valsa canker disease; however, the actual control effect is not ideal [5,6]. There are abundant mycovirus resources in fungi, some of which have become important biocontrol measures for controlling plant diseases caused by pathogenic fungi with attenuated virulent mycoviruses [7]. However, there are relatively few research reports on mycoviruses of Valsa canker pathogenic fungi in fragrant pears. The identification of mycoviruses in Valsa canker pathogenic fungi in fragrant pears in Xinjiang and the screening of attenuated virulent strains with biocontrol potential have important theoretical and practical significance for the prevention and control of Valsa canker disease in fragrant pears in Xinjiang.

Mycoviruses were initially discovered in *Agaricus bisporus* and subsequently identified in more than 100 species of fungi, including *Sclerotinia sclerotiorum*, *Botrytis cinerea*, *Rhizoctonia solani*, *Colletotrichum acutatum*, *Ustilaginoidea virens*, *Magnaporthe oryzae*, *Erysiphe trifoliorum*, etc. [8,9,10,11,12,13,14]. Mycoviruses can infect fungi and replicate in fungal cells and are transmitted mainly horizontally and vertically through hyphal fusion and spores [15,16]. Some mycoviruses not only affect the host’s life functions but also enhance the host’s defense capabilities [17,18]. Cryphonectria hypovirus 1 (CHV1) has been successfully used to control chestnut blight caused by *Cryphonectria parasitica* in Europe [19]. Rosellinia necatrix megabirnavirus 1 and Sclerotinia sclerotiorum hypovirulence-associated DNA virus 1 also have better control effects on *Rosellinia necatrix* and *Sclerotinia sclerotiorum*, respectively [20,21,22]. Recent studies have shown that some mycoviruses can convert pathogenic fungi into beneficial endophytes and activate plant immunity [23,24]. However, whether there are beneficial mycovirus resources that can be used for the prevention and control of Valsa canker disease in fragrant pears in Xinjiang still needs further exploration and analysis.

In recent years, many mycoviruses have been identified using high-throughput sequencing technology [25,26]. A total of 66 novel virus genomes were found in *Colletotrichum truncatum*, *Macrophomina phaseolina*, *Diaporthe longicolla*, *Rhizoctonia solani*, and *S. sclerotiorum* using macrovirus metagenomic sequencing analysis [26]. Approximately 14% of the *R. necatrix* strains from the Mediterranean region were infected by RNA viruses, and many of them were mixed infections [27]. A total of 283 new RNA viruses were identified in grape powdery mildew samples through metagenomic sequencing, and a new class of naked viruses was identified [28]. In previous studies, CHV1 and Mycoreovirus 1 (MyRV1) were introduced into *V. mali* via protoplast fusion, and both CHV1 and MyRV1 could be stably infected with *V. mali*, resulting in a reduction in fungal vegetative growth and virulence [29]. It is possible to obtain virus resources with good application potential by identifying viruses in *Valsa* spp. strains from the main pear-producing regions of Xinjiang.

In this study, 134 strains of *Valsa* spp. were isolated from diseased fragrant pear trees in Xinjiang, and the virome was investigated via metatranscriptomic sequencing. The mycoviruses in each *Valsa* spp. strain were identified using BLAST and RT-PCR. Furthermore, the impacts of the mycovirus on the growth and pathogenicity of *Valsa* spp. were also analyzed. The results of this study may lead to new ideas and biocontrol resources for the prevention and control of fragrant pear Valsa canker in Xinjiang.

## 2. Materials and Methods

### 2.1. Isolation and Identification of Pathogens

From March to June 2023, diseased branches or tree bark of fragrant pear were collected from Korla, Aksu, and Tumxuk in southern Xinjiang, China. A total of 199 isolates were isolated and purified from samples through tissue separation and single-spore purification. The strains were subsequently identified via morphological and molecular biological methods. A total of 199 isolates were cultured on potato dextrose agar (PDA) at 25 °C in a constant-temperature incubator and stored in 25% glycerol at −80 °C. The colony morphology was recorded after 5 days.

### 2.2. DNA and RNA Extraction

The total RNA of the *Valsa* spp. strains was extracted by using a total RNA isolation kit (Sangon Biotech, Shanghai, China) according to the manufacturer’s instructions and subsequently treated with DNase I to remove DNA contamination (Table S1). Fungal genomic DNA was extracted using the CTAB method [30]. The quality of the RNA and DNA was determined via agarose gel electrophoresis, and the total RNA and DNA samples were quantified via ultramicrospectrophotometry (NanoDrop 2000, Themo, Shanghai, China).

### 2.3. Metatranscriptomic Sequencing and Bioinformatics Analysis

According to the variations in growth rate and colony morphology, the *Valsa* spp. strains were divided into 5 groups labeled A, B, C, D, and E. There were 25 strains in each group (A–D), and 34 strains were in group E. Approximately 12,500 ng of RNA from each group was used for metatranscriptomic sequencing analysis performed using the Illumina MiSeq 2500 platform (Beijing Novogene Bioinformatics Technology Co. Ltd., Beijing, China). The Epicenter Ribo-ZeroTM Kit (Epicentre, Madison, WI, USA) was utilized for the removal of sample rRNA, the addition of fragmentation buffer to induce random interruption of RNA-depleted RNA, and the synthesis of the first cDNA strand using random hexamers. The second cDNA chain was synthesized by adding a buffer solution containing dATP, dUTP, dCTP, and dGTP along with RNase H and DNA polymerase I. The resulting cDNA library was purified using AMPure XP beads (Merck, Darmstadt, Germany) and further enriched through PCR amplification. IDBA_ud software was employed for the de novo assembly of clean reads (K = 19, 29, 39, 49, 59, 69, 79, 89, 99, 109, and 119) to generate contigs. Initially, the contigs were aligned against the NCBI rRNA, tRNA, and SILVA databases to separate sequences corresponding to rRNA, tRNA, snRNA, etc. A BLAST comparison against the Nr (NCBI non-redundant protein sequences) database was performed with an e-value threshold of ≤1 × 10^−3^. The Lowest Common Ancestor (LCA) algorithm was applied to determine the taxonomic annotations by considering the classification level before the first branch appeared in each sequence. According to the initial classification results, contigs belonging to the viral branch were chosen for BLASTx comparison on NCBI to refine the details of the virus, including the type and species of the viral nucleic acid. Homologous virus information was retrieved via BLASTp based on NCBI data. Contigs with more than 90% similarity to known viruses were considered to belong to different strains of the viruses.

### 2.4. Putative Mycovirus Sequence Confirmation

The RNA of *Valsa* spp. strains was used as a template to synthesize cDNA by using the EasyScript One-Step gDNA Removal and cDNA Synthesis SuperMix reverse transcription kit (TransGen, Beijing, China) with Oligo(dT)_18_ primers following the manufacturer’s instructions. The putative viral cDNA sequences were identified via RT-PCR using specific primers (Table S2) designed using Premier 5.0. The reaction system for RT-PCR was composed of 10 µL of 2× Taq Master Mix, 1 µL of upstream primer, 1 µL of downstream primer, 1 µL of cDNA, and ddH_2_O supplemented with 20 µL.

### 2.5. Phylogenetic Analysis

The BLASTX program was used to retrieve nucleotide sequences from the NCBI virus database. The multiple sequence alignments were aligned using MEGA 11.0 ClustalW as implemented in BioEdit 7.0. The aligned multiple sequences were used to construct a phylogenetic tree based on the maximum likelihood method using software (version MEGA11.0). The bootstrap values were set to 1000 replicates.

### 2.6. Growth Rate and Virulence Assay

Mycelial agar plugs (diameter: 5 mm) were selected from the colony edges of each strain cultured for 5 d and placed on PDA plates (diameter, 9 cm), which were subsequently incubated at 25 °C for 5 d in the dark to determine the mycelial growth rate and observe colony morphology. The virulence of each strain was determined by inoculating detached branches. Briefly, branches were inoculated with agar plugs of actively growing mycelia and then placed in a styrofoam chamber that was subsequently covered with a plastic membrane to maintain a constant humid atmosphere (90% relative humidity) at 25 °C in a constant-temperature incubator. Noncolonized PDA plates were also inoculated and incubated in parallel with the controls. The lesions that developed from the inoculated branches were measured and photographed at 15 d.

### 2.7. Horizontal Transmission of Hypovirulence Traits

The strains containing viruses (VN-5 infected with PlHV and CHV3; VN-34 infected with PlHV) and the virus-free strains (VN-VF: VN-10 and VS-9) were successfully isolated from diseased pear branches collected in Xinjiang, China. The activated donor strains were inoculated onto a PDA plate positioned approximately 1.5 cm away from the edge of the Petri dish and cultured for 2 days. Then, the recipient strain was placed 1.5 cm away from the donor strain and cultured in the dark at 25 °C, with the separately cultured strain serving as the control group. Three replicates were set for each treatment, and the experiment was repeated twice. The VN-34 and VN-5 strains were co-cultured with VN-FV in Petri dishes (diameter, 9 cm) at 25 °C for 10 d. After incubation of the contact cultures, mycelial agar plugs from the colony edge of the VN-FV strain were placed on fresh PDA plates, and four derived isolates were obtained from each recipient strain in the contact cultures. The derived strains were cultured on PDA plates layered with cellophane for 4 d, after which RNA was extracted for viral detection via RT-PCR.

## 3. Results

### 3.1. Isolation and Identification of Fragrant Pear Canker Disease Pathogens

**Figure 1:**
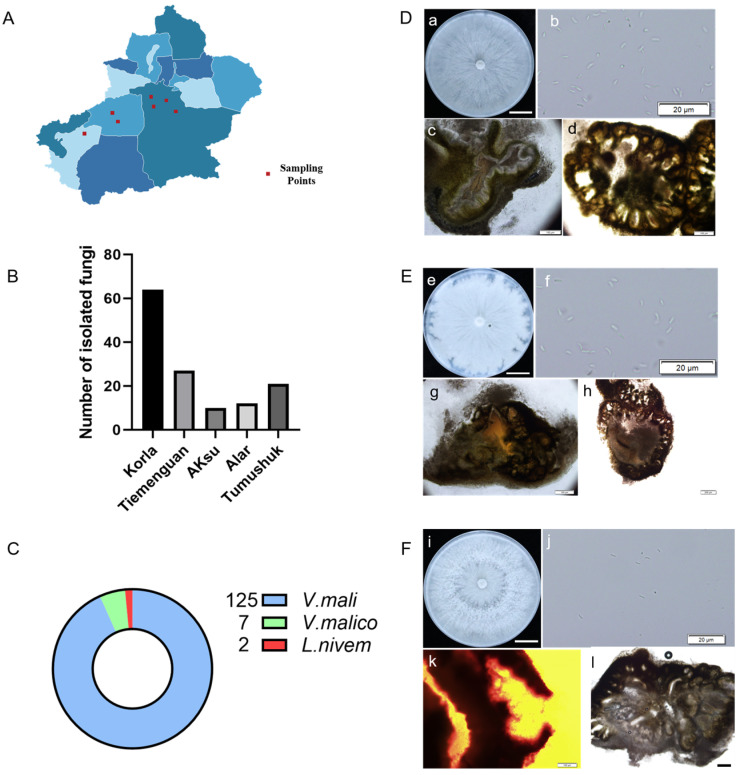
Information on the collection of canker disease samples and identification of pathogens: (**A**) the sample collection site; (**B**) the regional distribution of the isolated strains; (**C**) the number of *Valsa* spp. identified in different regions; (**D**) the morphological characteristics of *V. mali* strains. (**a**) Colony characteristics of *V. mali* on PDA media (bars = 2 cm); (**b**) the conidiospore of *V. mali* (bars = 20 μm); (**c**) the longitudinal section of conidiophore (bars = 100 μm); (**d**) the cross-section of conidiophore (bars = 100 μm); (**E**) the morphology of *V. malico* strains. (**e**) The colony characteristics of V. *malico* on PDA medium (bars = 2 cm); (**f**) the conidiospore of *V. malico* (bars = 20 μm); (**g**) the longitudinal section of conidiophore (bars = 100 μm); (**h**) the cross-section of conidiophore (bars = 200 μm); (**F**) the morphology of *L. niveum* strains. (**i**) the colony characteristics of *L. niveum* on PDA medium (bars = 2 cm; (**j**) the conidiospore of *L. niveum* (bars = 20 μm); (**k**) the longitudinal section of conidiophore (bars = 100 μm); (**l**) the cross-section of conidiophore (bars = 200 μm).

In the main production areas of fragrant pears in Xinjiang, a total of 199 unidentified strains were isolated from 300 samples (Figure 1A,B). Among these, 134 strains were preliminarily identified as *Valsa* spp. based on their morphological characteristics; these strains included 125 strains of *Valsa mali* (Figure 1C,D), 7 strains of *V. malicola* (Figure 1 C,E), and 2 strains of *Leucostoma niveum* (Figure 1C,F). The colony of *V. mali* was densely packed and appeared white or milky white (Figure 1D). The colony of *V. malico* was compact and white or milky white but had irregular edges (Figure 1E). The colony of *L. niveum* was dense and characterized by well-developed air mycelia (Figure 1F). These results are consistent with the molecular identification results obtained by using specific primers (Figure S1). *V. mali* was the dominant species, which was corrected to occupy 93%, and the maximum number of *Valsa* strains isolated from Korla was 65 (Figure 1B,C).

### 3.2. Metatranscriptomic Identification of Mycoviruses Infecting the Tested Strains

Illumina sequencing generated more than 52 Gb of raw reads, and 42.08 Gb of high-quality sequence reads were generated. A total of 41,180,587 clean reads were generated by removing the joints and inferior quality reads. The contigs obtained were blasted on NCBI, and the 19 contigs associated with viruses were screened out. The contigs were organized and assigned numerical labels based on a previous metatranscriptomic-sequencing-based grouping of strains. According to the results of comparison with the NCBI database, the results of amino acid sequence identification for Bcontig1, Ccontig6, Acontig4, and Bcontig33 were highly similar to those for Phomopsis longicolla hypovirus YP_009051682.1 and Fusarium asiaticum fusarivirus 1 UNG4436.1, Cytospora ribis mitovirus 1 AIS37555.1, and Magnaporthe oryzae narnavirus 1 BCH36657.1, respectively (Table 1). Consequently, these contigs were subsequently designated Phomopsis longicolla hypovirus (PlHV), Fusarium asiaticum fusarivirus 1-XJ1 (FaFV-XJ1), Cytospora ribis mitovirus 1 (CrMV1), and Magnaporthe oryzae narnavirus 1-XJ1 (MoNV-XJ1). The consistency of the amino acid sequences identified for Acontig5, Acontig11, and Ccontig35 was less than 90% for Cryphonectria hypovirus 3 NP_051710.1, Botourmiaviridae sp. UJQ91985.1, and Fusariviridae sp. WAK72331.11(Table 1), respectively, which were designated as novel viruses named Cryphonectria hypovirus 3-XJ1 (CHV3-XJ1), Botourmiaviridae sp.-XJ1 (BVsp-XJ1), and Fusariviridae sp.-XJ1 (Fvsp-XJ1).

### 3.3. Mycovirus Detection and Its Effect on the Growth of Valsa

**Figure 2:**
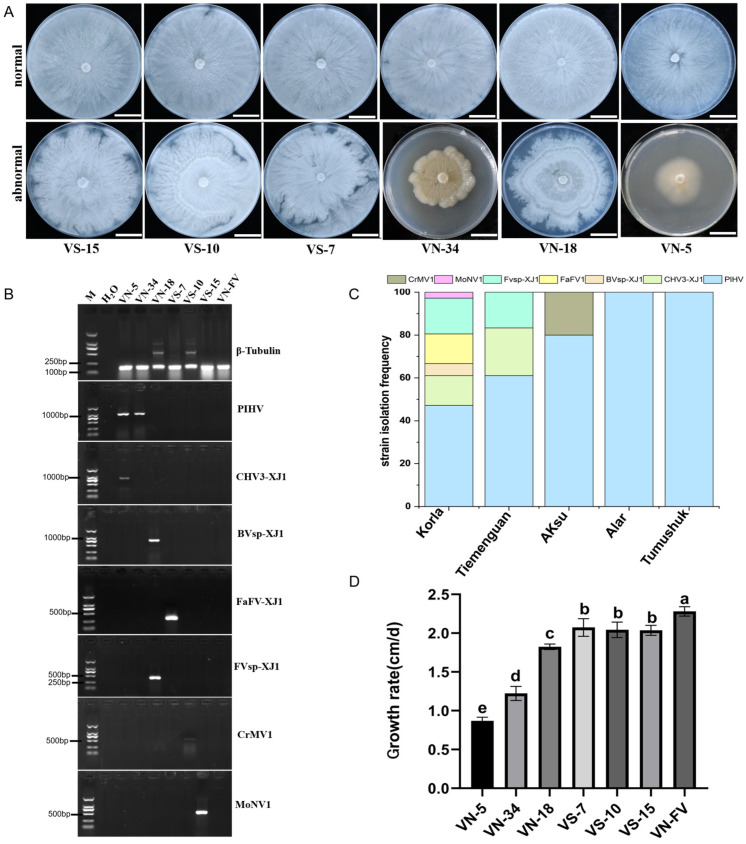
Detection of the seven mycoviruses and their effect on the growth of *Valsa* spp. (**A**) Colony morphology of *Valsa* spp. strains grown on PDA plates for 5 d (bars = 2 cm); (**B**) the detection of mycovirus contigs in different *Valsa* spp. strains. The internal reference gene β-Tubulin from *Valsa* spp. was used as the positive control, and ddH_2_O was used as the negative control. (**C**) Frequency of mycoviruses identified in different regions; (**D**) the growth rates of *Valsa* spp. strains harboring mycoviruses. The data are presented as means ± SD (*n* = 4). The different letters indicate a significant difference at *p* < 0.01 (determined via one-way ANOVA).

The distributions of the seven identified mycoviruses were analyzed via specific primers. The results showed that mycoviruses were present in all the sample collection areas and mainly distributed in Korla (Figure 2C). Interestingly, compared with those of the virus-free strains, the colony morphologies of the strains infected with the mycoviruses were abnormal, with irregular colony margins (Figure 2A,B), and the growth rate was slower for these strains than that for VN-FV (Figure 2D).The growth rate of the VN-5 strain infected with PlHV and CHV3-XJ1 was the slowest, followed by that of the VN-34 strain infected with PlHV, and infection with other mycoviruses (Bvsp-XJ1, Fvsp-XJ1, MoNV1, and FaFV-XJ1) also significantly slowed the growth rate of the hosts (Figure 2D).

### 3.4. Viral Sequence Phylogenetic Tree Analysis

**Figure 3:**
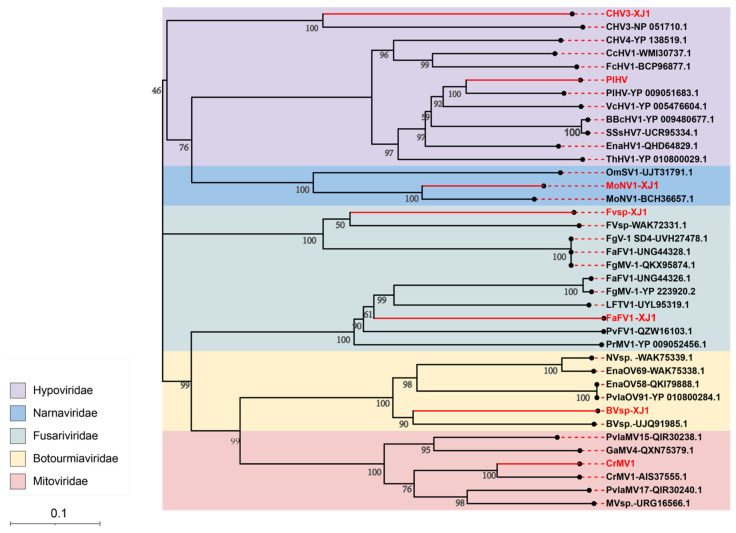
The phylogenetic tree of the mycoviruses in *Valsa* spp. based on RdRp amino acid sequences. A maximum-likelihood tree was constructed using MEGA 11.0 with 1000 bootstrap replicates. Bootstrap values (%) obtained with 1000 replicates are indicated on the branches, and the branch lengths correspond to genetic distance. The scale bar on the lower left corresponds to genetic distance. The resulting phylogenetic tree was exported using iTOL and Adobe Illustrator 2021 software. The viruses identified in this study are shown in red.

A phylogenetic tree was constructed based on the RNA-dependent RNA polymerase (RdRp) sequences of the identified mycoviruses. The results showed that PlHV and CHV3-XJ1 have close genetic relationships with CHV3 (NP051710.1) and PlHV (YP009051683.1), respectively, in the Hypoviridae group (Figure 3). FaFV1-XJ1 and Fvsp-XJ1 are classified into distinct branches in the Fusariviridae group and were identified as novel members of this viral family. Furthermore, BVsp-XJ1, CrMV1, and MoNV1-XJ1 were closely related to BVsp-XJ1 (UJQ919851.1), CrMV1 (AIS37555.1), and MoNV1 (BCH36657.1) in the Botourmiaviridae, Mitoviridae, and Narnaviridae families, respectively (Figure 3).

### 3.5. Hypovirulence Associated with PlHV and CHV3-XJ1

**Figure 4:**
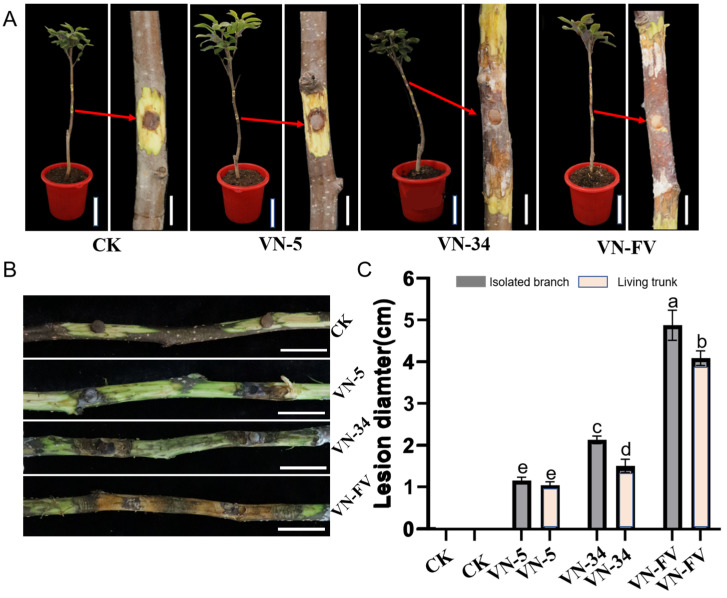
Pathogenicity assay of VN-5 and VN-34 on the living branches and detached branches. (**A**) The lesions of the VN-5, VN-34, and VN-FV strains on the stems of 2-year-old fragrant pear seedlings at 15 d, The arrow indicates the magnified view of the afflicted site. (bars = 10 cm and 2 cm in the pot and stem, respectively); (**B**) the lesions of the VN-5, VN-34, and VN-FV strains on the detached fragrant pear branches at 15 d (bars = 2 cm); (**C**) the lengths of lesions caused by VN-5, VN-34, and VN-FV strains on living trunk and detached branches of fragrant pear were assessed at 15 d. CK was inoculated with noncolonized PDA plugs. The data are presented as means ± SD (*n* = 4). The different letters indicate a significant difference at *p* < 0.01 (determined via one-way ANOVA).

In order to analyze the effect of PlHV and CHV3-XJ1 on the pathogenicity of the host, the pathogenicity of the *Valsa* spp. strains was determined via in vitro and in vivo inoculation. In addition to inhibiting host growth, PlHV and CHV3-XJ1 also affect host pathogenicity (Figure 4A,B). The pathogenicity of VN-5 infected with PlHV and CHV3-XJ1 and VN-34 infected with PlHV on detached and living branches was significantly reduced, resulting in smaller lesions than those on the control (Figure 4A,B). The lesion diameter of VN-5 was the smallest among both detached and living branches, with lesion diameters of 1.5 cm and 1 cm, respectively, representing decreases of 68.6% and 75%, respectively, compared with those of the control (Figure 4C), followed by VN-34 with lesion diameters of 2.2 cm and 1.8 cm, respectively, representing decreases of 54.2% and 55%, respectively, compared with those of the control (Figure 4C).

### 3.6. Horizontal Transmission of PlHV and CHV3-XJ1

**Figure 5:**
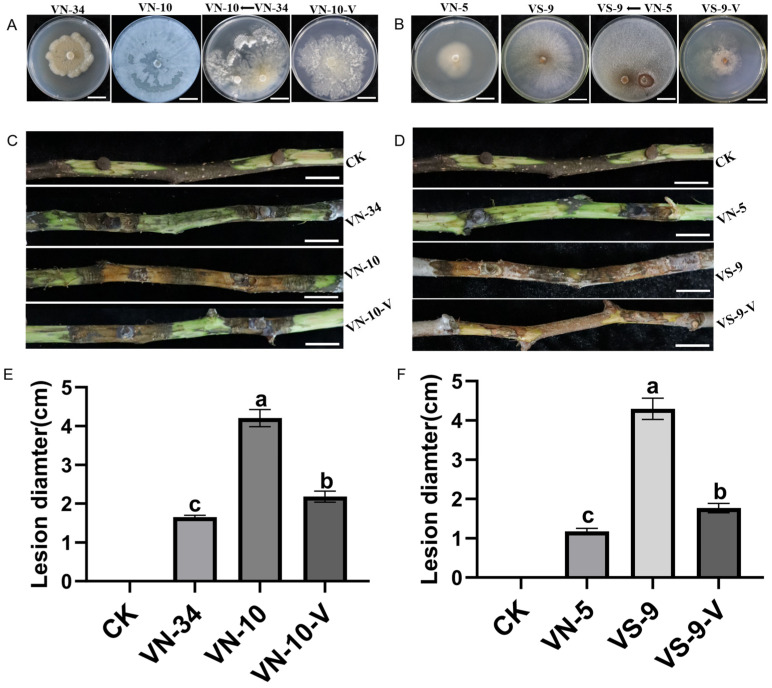
Horizontal transmission of PlHV and CHV3-XJ1 and their effect on the pathogenicity of the host: (**A**) colony morphology of the VN-10-V strain that acquired PlHV from VN-34 (bars = 2 cm); (**B**) colony morphology of the VS-9 strain that acquired PlHV and CHV3-XJ1 from VN-5 (bars = 2 cm); (**C**) the lesions of the VN-34, VN-10, and VN-10-V strains on the detached fragrant pear branches at 15 d (bars = 2 cm); (**D**) the lesions of the VN-5, VS-9, and VS-9-V strains on the detached fragrant pear branches at 15 d (bars = 2 cm); (**E**) the lesion diameters of the VN-34, VN-10, and VN-10-V strains on the detached fragrant pear branches at 15 d.; (**F**) the lesion diameters of the VN-5, VS-9, and VS-9-V strains on the detached fragrant pear branches at 15 d. CK was inoculated with noncolonized PDA plugs. The data are presented as means ± SD (*n* = 4). The different letters indicate a significant difference at *p* < 0.01 (determined via one-way ANOVA).

PlHV was successfully transferred from *V. mali* strain VN-34 to the virus-free *V. mali* strain VN-10 via co-culturing, for a transmission efficiency of 67% (Figure S2A), and the growth of VN-10 was also inhibited after transmission with PlHV (Figure 5A). Similarly, the growth of VN-9 was also inhibited after transmission with PlHV and CHV3-XJ1 (Figure 5B). After acquiring the mycoviruses, the pathogenicity of VN-10-V and VS-9-V on detached branches was significantly attenuated, resulting in smaller lesions than those on the control (Figure 5C,D). The lesion of the VS-9-V group was the smallest, with a lesion diameter of 1.77 cm (Figure 5F), followed by that of the VN-10-V group, with a lesion diameter of 2.18 cm (Figure 5E), representing decreases of 59% and 48%, respectively, compared with those of the control group (Figure 5F).

## 4. Discussion

With the advancement of bioinformatics, high-throughput sequencing technology has been regarded as the most efficient method for detecting and determining the evolution of viruses [7,31,32,33]. The identification of mycoviruses in pathogenic fungi is helpful for the exploration of mycovirus resources with biocontrol potential [7]. Several mycoviruses with great biocontrol potential have been successfully identified and applied in plant disease management. For example, CHV1 and SsHADV1 have better control effects on chestnut blight and oilseed rape sclerotinia rot, respectively [34,35]. Our results showed that a total of seven mycoviruses distributed in five families were identified among 134 *Valsa* spp. strains via metatranscriptomic sequencing. Three new mycoviruses were discovered, and two hypovirulence strains harboring mycoviruses showed great biocontrol potential. The results of this study not only clarify the species and distribution of mycoviruses in the canker disease pathogen of fragrant pear in Xinjiang but also provide biocontrol resources for the prevention and control of canker disease.

The growth and development of fungi are often inhibited by mycoviral infection, the morphology of which also changes accordingly because the signaling and metabolic pathways of the hosts are usually disrupted by mycoviruses [36,37]. For example, the growth rate of the *S. sclerotiorum* SZ-150 strain coinfected with Sclerotinia sclerotiorum hypovirus 1 and its related satellite RNA slowed, and the colony edge was abnormal [38]. The growth rate of the *S. sclerotiorum* AHS31 strain was reduced after being coinfected with botourmiaviruses and mitoviruses, and its pathogenicity decreased significantly [39]. Fungal infection with PlHV results in various phenotypes, ranging from a normal phenotype to stunted colonies with irregular colony edges [40], which is consistent with the results of the present study. Infection with PlHV not only affected the colony morphology of *V. mali* but also showed certain biocontrol potential because the pathogenicity of VN-34 after infection with PlHV seriously decreased.

After multiple mycoviruses infect a single strain, the direct or indirect interactions between the mycoviruses are very complex and involve synergistic and antagonistic effects. The Sclerotinia sclerotiorum mycoreovirus 4 (SsMYRV4) acts as a “bridge” for promoting the transmission of heterologous mycoviruses (such as SsDRV and SsMV1) in the *S. sclerotiorum* population because SsMYRV4 can inhibit the expression of heterotrimeric guanine nucleotide-binding proteins and some vegetative-compatibility-related genes [41]. The yado-kari virus 1 (YkV1), without encoding capsid protein, can hijack the capsid protein of the yado-Nushi virus 1 (YnV1) to enhance its survival when it is co-infected with *R. necatrix* [42]. In addition to affecting the growth of *C. parasitica*, it was observed that CHV3 also had synergistic effects on *C. parasitica* [43] through further reducing the influence of PlHV on the pathogenicity of *V. mali* in this study. However, the synergistic mechanism of PlHV and CHV3-XJ1 is unclear, so further study is needed.

Botourmiaviridae viruses were initially found in plants and recently detected in fungi such as *Botrytis* spp. [43] and *Pyricularia oryzae* [42], expanding their host range beyond the plant kingdom. These viruses may be transmitted from plant to pathogenic fungi through infection by pathogens [41]. Our study also detected Botourmiaviridae viruses and found that they had no significant effects on the growth or virulence of *V. mali*, which is consistent with previous research results [44,45]. On the other hand, Fusariviridae viruses have the ability to infect diverse plant pathogens, including *Nigrospora oryzae* [46], and *Neofusicoccum luteum* [47], etc. [48,49,50], and inhibit the mycelial growth and pathogenicity of *Fusarium tricinctum* [50,51]. However, in addition to affecting the mycelial growth of *V. mali*, the Fusariviridae viruses did not affect the pathogenicity of *V. mali* in the present study, indicating that there were different mechanisms of action of Fusariviridae viruses in different hosts.

The transmission characteristics of mycoviruses play an important role in these viruses’ application [29,41]. In addition to inhibiting the pathogenicity of *V. mali*, PlHV also had a high horizontal transmission efficiency (67%) in the present study. These findings indicate good biocontrol potential against canker disease. Interestingly, when *V. mali* strain VN-5 was co-cultured with other strains, the transmission efficiencies of PlHV and CHV3-XJ1 in VN-5 were low, amounting to 25% and 17%, respectively. We speculated that CHV3-XJ1 may inhibit the transmission of PlHV. Yang et al. reported that the transmission efficiency of CHV1 was reduced by activating *dcl2* expression in the host when *V. mali* was coinfected with CHV1 and MyRV [29]. Whether the decreases in the transmission efficiency of PlHV and CHV3-XJ1 are due to the same mechanism of action requires further study.

The identification of mycoviruses in pathogenic fungi is helpful for screening beneficial biocontrol resources. Many partitiviruses and chrysoviruses have been identified in phytopathogenic fungi, some of which are involved in the hypovirulence of their hosts [52,53]. To the best of our knowledge, this is the first study to systematically identify mycoviruses in *V. mali* from fragrant pear canker disease in Xinjiang, China. More importantly, two hypovirulent *V. mali* strains harboring mycoviruses were identified and showed good application potential. The results of the present study provide a scientific basis for future research on the development of mycoviruses as biological control agents for Valsa canker disease in the field.

## Figures and Tables

**Table 1:** Best BLASTx matches of the contigs obtained.

Number	Contig Number	Contig Length	Name of Putative Viruses	Best Match	aa Identity	Genome Type	Family/Genus
1	Bcontig1	9810	Phomopsis longicolla hypovirus (PlHV)	Phomopsis longicolla hypovirus YP_009051683.1	99%	+SSRNA	*Hypoviridae*
2	Acontig5	2969	Cryphonectria hypovirus 3-XJ1(CHV3-XJ1)	Cryphonectria hypovirus 3 NP_051710.1	81%	+SSRNA	*Hypoviridae*
3	Bcontig11	2519	Botourmiaviridae sp.-XJ1(BVsp-XJ1)	Botourmiaviridae sp. UJQ91985.1	83%	+SSRNA	*Botourmiaviridae*
4	Ccontig6	6046	Fusarium asiaticum fusarivirus 1-XJ1(FaFV1-XJ1)	Fusarium asiaticum fusarivirus 1UNG44326.1	98%	+SSRNA	*Fusariviridae*
5	Ccontig35	653	Fusariviridae sp.-XJ1(Fvsp-XJ1)	Fusariviridae sp. WAK72331.1	57%	+SSRNA	*Fusariviridae*
6	Acontig4	3046	Cytospora ribis mitovirus 1 (CrMV1)	Cytospora ribis mitovirus 1 AIS37555.1	100%	+SSRNA	*Mitoviridae*
7	Ccontig33	665	Magnaporthe oryzae narnavirus 1-XJ1(MoNV1-XJ1)	Magnaporthe oryzae narnavirus 1BCH36657.1	98%	+SSRNA	*Narnaviridae*

## Data Availability

The data presented in this study are available in the article.

## Supplementary Materials

The following supporting information can be downloaded at https://www.mdpi.com/article/10.3390/v16030355/s1, Figure S1: Molecular identification of 134 *Valsa* spp. strains; Figure S2: Detection of horizontal transmission of PlHV and CHV3-XJ1; Table S1: The specific primers of *Valas* spp.; Table S2: The primers used to detect mycoviruses in this study.
