# Supplementary material for: Identification of Mycoviruses in the Pathogens of Fragrant Pear Valsa Canker from Xinjiang in China
Source: Viruses. 2024 Feb 25;16(3):355. doi: 10.3390/v16030355 (PMC10974596; doi:10.3390/v16030355)
Supplement: Supplementary file 1 [file viruses-16-00355-s001.zip › viruses-2872232-supplementary.pdf]

Supplementary Materials

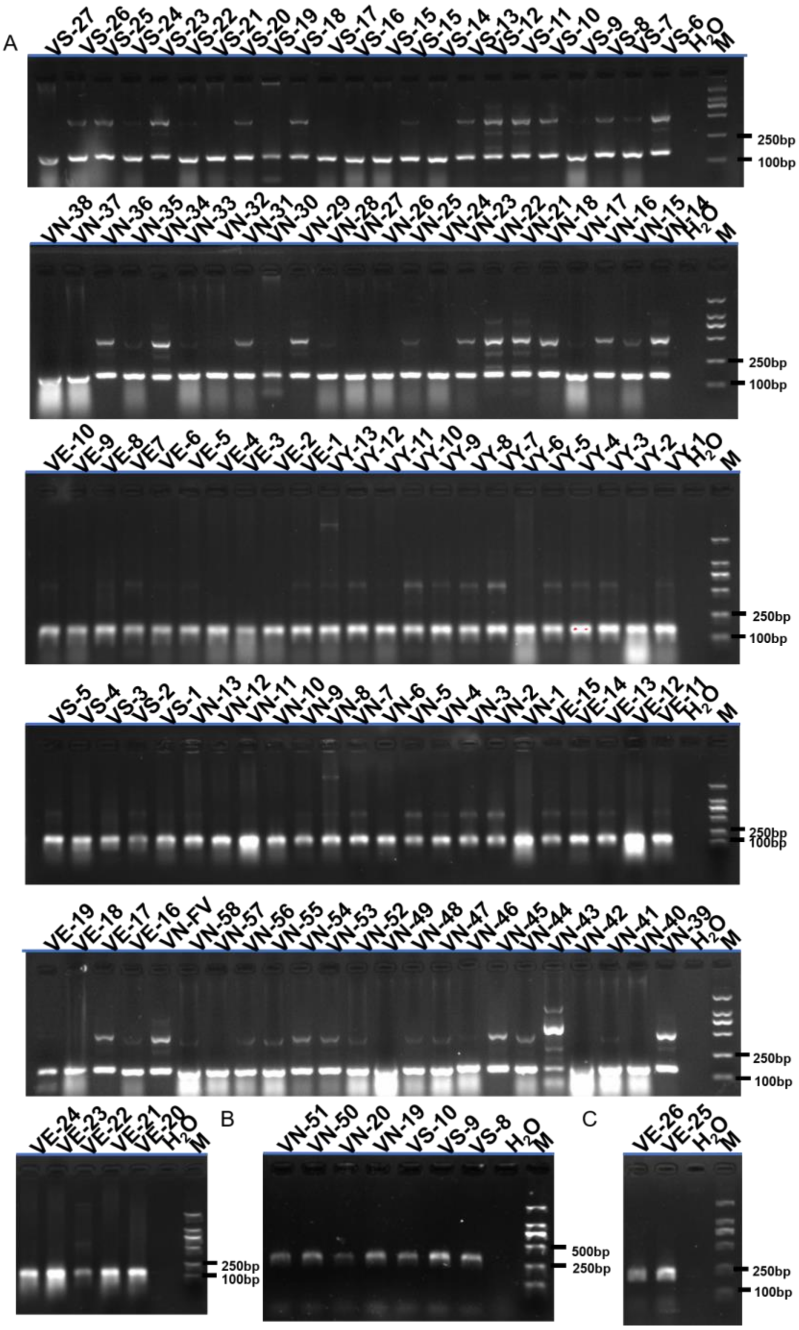

Figure S1. Molecular identification of 134 *Valsa* spp. strains. (A) The identification of *V. mali* strains by using the specific primers of *V. mali*. The expected fragment size was 144bp. (B) The identification of *V. malico* strains by using the specific primers of *V. malico*. The expected fragment size was 324bp. (C) The identification of *L. niveum* strains by using the specific primers of *L. niveum*. The expected fragment size was 240bp.

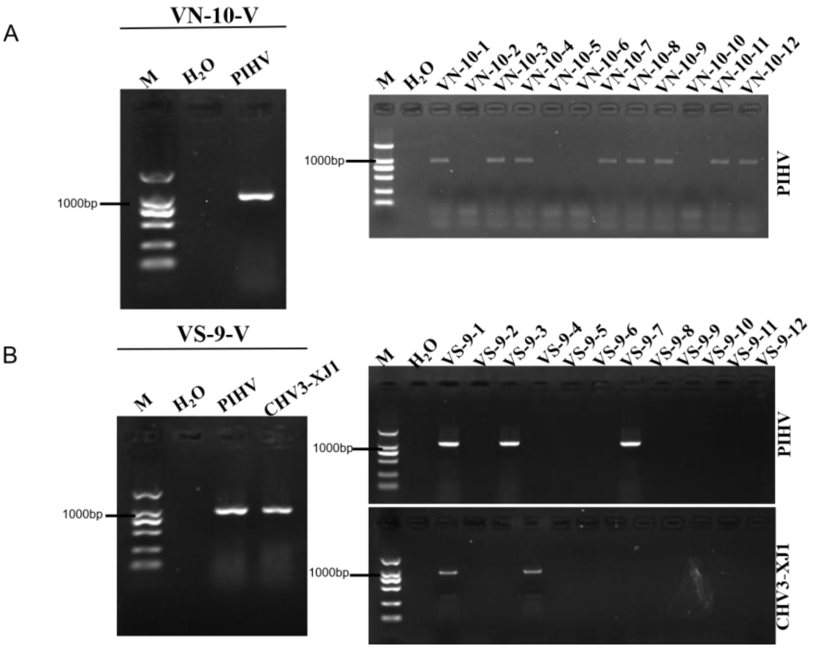

Figure S2. Detection of horizontal transmission of the PHIV and CHV3-XJ1. (A) Detection of the PIHV in the derivative isolate strain VN-10-V. (B) Detection of the PHIV and CHV3-XJ1 in the derivative strain VS-9-V.

Table S1. The specific primers of *Valas* spp.

V. species	Primer name	Primer Sequence (5′-3′)	Annealing temperature (℃)
V. mali	VcF	ATTGTGCGATGGCCATCATCTCG	52
	VcR	CAAGGCTAGAAGCAACGCGAAA	59
V. sordida	VsF	CGCAACCCATCTTTCGCTTC	50
	VsR	CTGGACGGAAACACGTCAGT	49
V. malicola	VmF	CGTACCTCCAACTCCATCCC	60
	VmR	CATACTTGTTGCCGGAAGCC	56
L. niveum	VnF	TTTCCTTCGCGTACAGGTCC	59
	VnR	GTTGTAACTGCGCGATGGTC	58

Table S2. The primers used to detect mycoviruses in this study.

species of virus	Primer name	Primer Sequence (5′-3′)	Annealing temperature (℃)
PlHV	1PlHV962F	GCTATTTGCACACTCTTCAC	55
	1PlHV962R	CCAATACAGAGTACATGCGA	55
CHV3-XJ1	2Chv3-834F	CTTCCTTTGCCAACTATCCT	55
	2Chv3-834R	CGGCAAGCAATATCATCATC	55
BVsp-XJ1	1Bvsp814F	CGGACGTACATGGTCTTAAT	55
	1Bvsp814R	CACTGAAAATTAGACGTGGC	55
FaFV1-XJ1	2Fafv1305F	GCAAAGACTAAGAACCCAGA	55
	2Fafv1305R	CCAGTGAATGTGTCTAACCA	55
Fvsp-XJ1	2Fvsp403F	ATTATTTGCCCGTCCTTGAT	55
	2Fvsp403R	TTGGCTCGTTCATCTACAAA	55
CrMV1	2Crmv1554F	GACTCTATGAGAGCAACAGG	55
	2Crmv1554R	ATTTGCGAACTAAGGTCTGT	55
MoNV1-XJ1	2Monv-472F	ATCACGACAGACCCTTACTA	55
	2Monv-472R	GATGTTCGTCTCTCCTTACC	55
